# Investigating the Impact of Guided Imagery on Stress, Brain Functions, and Attention: A Randomized Trial

**DOI:** 10.3390/s23136210

**Published:** 2023-07-07

**Authors:** Katarzyna Zemla, Grzegorz Sedek, Krzysztof Wróbel, Filip Postepski, Grzegorz M. Wojcik

**Affiliations:** 1 Institute of Psychology, SWPS University of Social Sciences and Humanities, 03-815 Warsaw, Poland; kzemla1@st.swps.edu.pl (K.Z.);; 2 Department of Neuroinformatics and Biomedical Engineering, Institute of Computer Science, Maria Curie-Sklodowska University, 20-033 Lublin, Polandfilip.postepski@mail.umcs.pl (F.P.)

**Keywords:** guided imagery, relaxation, stress reduction, cognitive performance, EEG, GLM

## Abstract

The aim of this study was to investigate the potential impact of guided imagery (GI) on attentional control and cognitive performance and to explore the relationship between guided imagery, stress reduction, alpha brainwave activity, and attentional control using common cognitive performance tests. Executive function was assessed through the use of attentional control tests, including the anti-saccade, Stroop, and Go/No-go tasks. Participants underwent a guided imagery session while their brainwave activity was measured, followed by attentional control tests. The study’s outcomes provide fresh insights into the influence of guided imagery on brain wave activity, particularly in terms of attentional control. The findings suggest that guided imagery has the potential to enhance attentional control by augmenting the alpha power and reducing stress levels. Given the limited existing research on the specific impact of guided imagery on attention control, the study’s findings carry notable significance.

## 1. Exploring the Impact of Relaxation Techniques on Brain Wave Activity and Attentional Performance: A Review of Relevant Research

Improving attention and executive functions is of great importance in our current world due to the complex and demanding nature of daily tasks and the challenges posed by our modern reality. Scientific research has shown that attention and executive functions play crucial roles in various aspects of cognitive processing and goal-directed behavior [1]. Attention is the cognitive process that allows us to selectively focus on relevant information while filtering out irrelevant stimuli [2]. It is essential for tasks that require concentration, information processing, and decision making. In our information-rich environment, where we are constantly bombarded with stimuli and distractions, the ability to maintain focused attention is vital for productivity and task performance. Scientific studies have consistently demonstrated the positive impact of enhanced attention and executive function on various aspects of individuals’ lives [3]. Improved attentional control and executive function have been associated with a better academic performance [4,5,6] and job performance [7] and professional success. Additionally, they contribute to effective decision making, problem solving, and conflict resolution. Hence, improving attention and executive functions is vital in our current world, given the cognitive demands and challenges we face, ultimately benefiting individuals and society as a whole in wide range of EEG experiments designed to quantitatively measure cognitive functions like those in [8]. In recent years, there has been a growing interest in studying the effects of meditation and relaxation techniques on attentional control processes. Tang [9] conducted a study that demonstrated how just five days of mindfulness meditation training improved attentional control in healthy young adults. Similarly, Zeidan [10] found that brief mindfulness meditation training improved executive attentional control abilities and reduced anxiety. Furthermore, Ruedy and Schweitzer [11] found that a brief period of relaxation exercises enhanced participants’ ability to resist distractions and maintain focus on a cognitive task. Several reviews have also analyzed the impact of meditation on cognitive functions, including attention, memory, and executive control. For instance, Chiesa and Serretti [12] examined the effects of mindfulness meditation on attentional control and found that it led to improvements in both selective and sustained attention. Many studies in cognitive psychology and neuroscience have explored the positive impact of mindfulness and meditation training on cognitive functions. These studies have utilized a wide range of tasks to assess measures of response accuracy, response time, and associated electrophysiological and neuroimaging patterns, highlighting the positive impact of mindfulness and meditation on cognitive performance [9,13,14,15,16,17].

Despite being recognized as a healing resource for centuries [18], the potential impact of guided imagery (GI) on cognitive performance remains largely unexplored. In recent years, there has been increased interest in the role of GI in health and well-being [19]. GI has also been found to be effective in enhancing sports performance [20]. In the late 1970s, health professionals reported using imagery for altering the course of life-threatening diseases [21,22]. Studies have shown that GI can reduce psychological stress and smoking behaviors among smokers and ex-smokers [23], and can facilitate improved health behaviors and reduce psychological distress in the workplace [23]. GI involves external instructional guidance to allow the internal generation of images [24], and it is defined as the mental process that employs the senses of sight, hearing, smell, and taste. Sensations of motion, position, and contact are experienced by GI practitioners [25]. GI has wide-ranging relevance and applicability, and is effective in reducing test anxiety [26], coping with stress [27,28,29,30,31,32,33,34,35], and improving problem-solving abilities [36].

Given the evidence of both anxiety reductions and immune system enhancements, GI has not been studied during brain behavior and brainwave changes while patients are conducting GI sessions. However, the ever-growing neuroscience literature relating to the phenomena of mindfulness sessions is trying to incorporate EEG quantitative measurements to describe brain wave changes during mindfulness sessions [37]. For example, in the research led by Peta Stapleton, the brainwave data of a group of 468 meditation novices with limited previous exposure to forms of guided meditation were recorded, and researchers observed a global increase of 16% (95% HDI = [0.13, 0.19]) in alpha power due to meditation [38]. A range of mindfulness-based techniques has been created to reduce stress and enhance the quality of life [39]. Meditation is a complex conscious cognitive process requiring concentration and receptive attention [9,40]. Meditation practices are also associated with enhanced executive function and working memory [41,42,43,44,45]. However, little research has provided an electrophysiological examination of the meditative experience in people with limited meditation experience, particularly from a GI perspective. However, it is known that alpha activity in EEG signals during meditation is a form of brain integration that leads to higher-level cognitive processes [46]. Researchers hypothesized that the transition from beta brainwaves (high, medium, and low range) to alpha brainwaves could take place relatively quickly [38]. This result is consistent with the findings of a study in which participants achieved proficiency in the attentional training aspect of meditation practice relatively swiftly [47]. An increase in alpha wave levels indicates that the participants are in a relaxed mood or their mood is enhanced [48]. Under stress, the alpha brain waves tend to decrease, which can indicate a state of heightened arousal and anxiety. The alpha frequency is also positively correlated with the speed of processing information [49]. On the other hand, the beta wave power indicates that humans are in an alert condition [50]. An increased beta activity can interfere with the ability to relax and can make it difficult to focus attention on a single task. Research has shown that stress can interfere with attention control by reducing our ability to filter out distractions and interfering with our ability to shift our focus from one task to another [51]. Zoefel proved [52] that an increase in EEG alpha wave activity is linked to an improvement in cognitive performance. Cognitive control (CC) and executive function (EF) are defined in relation to goal-directed behavior versus habits and controlled versus autonomic processing, as well as the functions of the prefrontal cortex (PFC) and associated regions and networks [53]. Executive functions (EFs) consist of a family of three, interrelated core skills: (1) inhibition or active suppression of stimuli and automatic responses that are irrelevant to the task at hand, (2) updating and monitoring of information in the working memory to include only the most relevant material, and (3) shifting or switching attention between multiple mental representations or operations [54].

Anti-saccade, Stroop, and Go/no-go tasks are three commonly used tests to assess executive function, which refers to a set of cognitive processes involved in goal-directed behaviors [55]. These tasks have been extensively studied and validated, allowing for meaningful comparisons across different studies and populations [56]. Although all three tests are measures of executive function, they differ in their specific cognitive demands and the underlying processes they assess. Anti-saccade tasks assess inhibitory control and attentional control [54,57], Stroop tasks assess selective attention and inhibition of irrelevant information, and Go/No-go tasks assess response inhibition and working memory [58]. It was proven that acute psychosocial stress may affect executive action control in a Go/No-go task [51].

No research was found on attentional tasks after GI sessions. However, it is known that other relaxation techniques such as meditation can reduce interference during the Stroop task [59], and meditators have better attentional performance in the Stroop task compared with a meditation-naïve control group [60]. High proficiency in this task indicates good attentional control and relatively low automaticity or impulsivity of one’s responses [13]. The study titled “Mindfulness-of-breathing exercise and its effect on EEG alpha activity during cognitive performance in an attentional Stroop task” investigates the relationship between a mindfulness-of-breathing exercise and EEG alpha activity during cognitive performance, specifically in the context of an attentional Stroop task [61]. The study results showed a significant increase in alpha power during the intervention among the mindfulness-of-breathing exercise group compared to the control group. The mindfulness-of-breathing exercise group also demonstrated a trend toward enhanced performance in the Stroop attentional blink task after the intervention. The authors suggest that the increased alpha power may potentially facilitate cognitive performance [61]. Another study “Short Term Integrative Meditation Improves Resting Alpha Activity and Stroop Performance” [62] provides evidence that a short-term integrative meditation program can improve the resting alpha activity and cognitive performance in the Stroop task. Another commonly used measure of cognitive inhibition is the anti-saccade (AS) task, which requires suppression of a visually guided saccade toward a target and the generation of voluntary saccades in the opposite direction. In was concluded in [57,63] that more accurate and more consistent AS performances were present in meditators in comparison to the non-meditators group. Go/No-go tasks can provide objective evidence of attention lapses in the form of target omission errors and response time variability. In [64], the authors reported that mindfulness is related to errors on Go/No-go tasks with high self-reported mindfulness scores are related to more accurate responses [60,65,66,67,68]. Inhibition, shifting, and updating are core abilities that support a mindful state and are facilitated via regular meditation [69]. For example, inhibition of unrelated mental representations and reactions is required to maintain a mindful state, with inhibitory control increasing once a mindful state is achieved via implicational intentions (e.g., if the mind is wandering, then disengage and refocus attention). Shifting is necessary to mentally clear distractions and unrelated representations back to the present-moment experience. Finally, updating the working memory is required to continually stay focused on an ever-changing present moment [70].

However, there is no research on GI and its impact on the results of attention tests. Only [71,72] verified and proved that relaxation induced by GI significantly enhanced working memory performance, but there is no other research on the topic that this research investigates.

## 2. The Potential Benefits of Guided Imagery for Executive Function and Attentional Control and Research Hypotheses

Guided imagery offers a distinct experiential approach to mindfulness and mental well-being. Although meditation primarily focuses on cultivating present-moment awareness and detachment from thoughts, guided imagery involves actively engaging the imagination to create vivid sensory experiences. This approach can be particularly helpful for individuals who find it challenging to quieten the mind or those who benefit from more structured practices. A further exploration of guided imagery is interesting as it broadens our understanding of mindfulness, offers customization, and provides a complementary practice to enhance mental health.

Overall, mindful meditation and GI practices can be effective for improving attention control and cognitive performance; however, the specific benefits and mechanisms of action differ depending on the practice. Mindfulness meditation develops greater awareness and control over the mind [73] and GI promotes positive emotions and reduces stress and anxiety, whereas anxiety impairs the cognitive performance by increasing cognitive interference [74]. Effective stress management strategies, such as relaxation techniques, may be helpful in mitigating the negative effects of stress on attention and cognitive functions. Attentional control theory posits that for goal-directed behavior to occur, attentional control is necessary, involving inhibiting competing demands to concentrate on the current task and being able to switch or shift attention as necessary [75]. Attentional control theory specifies that deficits in these aspects of attentional control are central to the development and maintenance of anxiety [75]. In support of this assumption, a recent meta-analysis of 58 studies testing the association between measures of attentional control and anxiety found that participants with high anxiety showed a deficit in attentional control compared to participants with low anxiety [76].

The main research hypothesis of this study was that a short-term GI session would reduce stress levels in healthy male participants without prior experience with such sessions or a history of chronic medical conditions. To test this hypothesis, 30 participants were randomly selected to undergo a GI session, and the effectiveness of the session in reducing stress was assessed through monitoring beta power reductions and alpha state increases using EEG data recordings and self-reported questionnaires.

In addition to evaluating the effectiveness of the GI session, this study aimed to investigate whether the results of attentional tasks (Stroop, Go/No-go, and anti-saccades tests) could differentiate between the group of participants who underwent the GI session and another group of 30 randomly selected male participants who completed a mental task. Specifically, the number of errors made on these tasks between the two groups was compared.

Furthermore, the study hypothesized that changes in alpha power might mediate the relationship between the utilization of GI and the decrease in errors on the Stroop and anti-saccade tests. To test this hypothesis, a mediation analysis was conducted to explore the possible relationship between these variables.

## 3. Materials and Methods

### 3.1. Materials

Before the experiment, the participants were required to sign a consent form confirming their willingness to participate. The participants were also required to fill in their personal information and answer several questionnaires as outlined below:

1. Scales of Helplessness and Anxiety of Contracting an Infectious Disease by Rydzewska, K. and Sędek, G. 2020 unpublished research materials from SWPS University of Social Sciences and Humanities. These measures were used to indicate the potential role of high levels of maladaptive emotions in impeding rational decision making during the pandemic.
2. The State-Trait Anxiety Inventory (STAI) is a self-reporting questionnaire designed to measure anxiety in adults. The STAI questionnaire is often used in medical and research settings to help identify people who may need treatment for anxiety [77]. It can also help to measure the effectiveness of treatments designed to reduce anxiety.
3. Following both the GI and mental task sessions, participants underwent attentional tests to test the hypothesis that GI can enhance attentional control.
  The anti-saccade test—attention control was designed according to the recommendations of the Antoniades protocol. In prosaccade trials, the object appears at the location of the cue, so the discrimination of stimuli is relatively easy. The primary indicator in this task is the average percentage of correct responses for the anti-saccade blocks.
  The numerical Stroop Test is a variation of the classic Stroop test that uses numbers instead of words. The test is designed to create interference between the automatic response of reading the digits and the task of counting them, which requires more cognitive effort. The test measures the ability to suppress automatic responses (response inhibition) and focus attention on the task at hand [78].
  The main indicator in this test is the average percentage of correct answers.
  Go/no-go tasks require participants to respond to one type of stimulus (the “go” stimulus) but inhibit their response to another type of stimulus (the “no-go” stimulus). This task assesses the ability to inhibit automatic responses and cognitive flexibility, as well as response inhibition and working memory [58]. The tasks in the main block were arranged in a pseudorandomized order while following the rule that No-go trials were preceded by two or five Go trials. The main block of trials was preceded by ten practice trials, consisting of two No-go and eight Go trials. As a primary measure of Go/No-go task performance, the attention control was the percentage of correct responses to Go trials after No-go trials.
4. Furthermore, both prior to and following the GI and mental task sessions, the study participants were given questionnaires developed by the research team. These questionnaires encompassed various measures, including participants’ self-reported levels of stress and relaxation on a 10-point scale, and enabled the identification of emotions experienced by the participants before and after the GI and mental tasks.

The experimental group underwent a recorded GI session, in which participants were provided with a series of instructions to visualize a calming and peaceful scenario. The session began with simple breathing exercises and progressive muscle relaxation techniques. The mental task group listened to a pre-recorded session consisting of mental tasks that involved recalling the names of voivodeships in Poland, zodiac signs, and other similar tasks. The inclusion of a mental task in the second group, rather than a resting state condition, was designed to simulate the experience of stress. Stress is known to elicit negative thoughts and worry, leading to cognitive rumination [79]. This repetitive thinking about stressors, problems, or potential threats can be mentally exhausting and hinders the ability to achieve a state of relaxation. The cognitive load associated with stress-related thoughts keeps the mind engaged, making it difficult to enter a restful state. Therefore, the use of a mental task was aimed to replicate the cognitive demands and stress-related cognitive processes often experienced in real-life stressful situations.

Both experimental groups, including the guided imagery group and the mental task group, were subjected to identical conditions, which involved listening to pre-recorded instructions for the same duration. Furthermore, each experimental session was supervised by two trained technicians who diligently attended to technical aspects, ensuring proper electrode placement and functioning, including the playback of the recordings.

To determine whether participants in the guided imagery and mental task group were actively engaged in the experiment and not sleeping, researchers employed several strategies to minimize the likelihood of participants falling asleep during the session summarized in the following. Monitoring: Researchers were present during the session and monitored participants during the guided imagery session and mental task session. This allowed to visually confirm whether participants remained awake and actively participated throughout the session. Instructions: Clear instructions were provided to participants before the guided imagery session and mental tasks session, emphasizing the importance of staying awake and engaged. Post-session debriefing: After the guided imagery session and mental task session, researchers conducted a debriefing via a survey with participants to ask about their experience and level of engagement. These measures, combined with the researchers’ direct observations and vigilance, can provide valuable evidence to ascertain whether participants in the guided imagery group remained awake and actively participated in the experiment. However, it is important to note that despite these efforts, it is challenging to completely eliminate the possibility of some participants unintentionally falling asleep during a session. However, the study conducted by Yaxin Fan “Short Term Integrative Meditation Improves Resting Alpha Activity and Stroop Performance” [62] provides evidence that, in contrast to the significant changes observed in the meditation training group, no significant alterations in alpha power or performance on attention tasks are observed even during a resting state in the control group.

### 3.2. Experimental Facilities

The EEG Laboratory located within the Department of Neuroinformatics and Biomedical Engineering is equipped with a dense array amplifier that can capture brain electrical activity at a frequency of 500 Hz using a 256-channel HydroCel GSN 130 Geodesic Sensor Net. This complete and compatible system is manufactured by Electrical Geodesic Systems, and it utilizes a Geodesic Photogrammetry System (GPS), which uses 11 cameras placed in its corners to create a model of the subject’s brain based on its size, proportion, and shape. This system is able to accurately superimpose computed activity results onto the brain model. The amplifier works in conjunction with the Net Station 4.5.4 software, while the GPS is controlled by Net Local 1.00.00 and GeoSource 2.0. Eye tracking was achieved through the use of a SmartEye 5.9.7 system, which allows for gaze calibration and the elimination of eye blinks and saccades. PST e-Prime 2.0.8.90 was used to design the ERP experiments.

### 3.3. The Cohort

The Bioethical Commission of Maria Curie-Sklodowska University in Lublin, Poland, granted permission for all the experiments described below. During the relaxation experiment, each participant in the cohort sat in a comfortable armchair with earphones and listened to a recording of a relaxation procedure. The recording was prepared by a trained expert using a typical method of GI, which as explained above in detail is a relaxation technique that involves focusing on a positive mental image or scene. The recording was 21 min and 7 s long, but for this research, only the first 20 min were considered. It was assumed that each member of the sub-cohort would eventually become relaxed enough to manifest brain cortical activity that could be classified.

**Figure 1:**
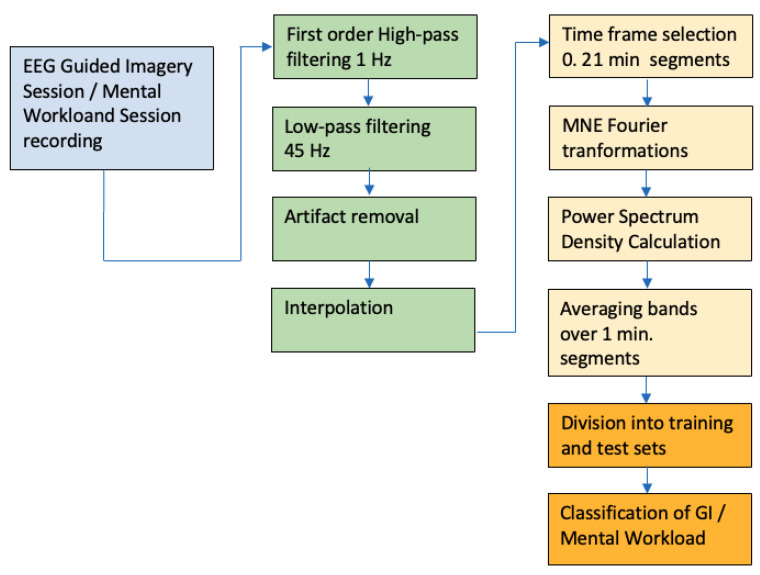
Research protocol used for data processing of both types of sessions: GI and mental task workloads.

We utilized our dense array amplifier to capture the signals across all 256 electrodes. However, considering our prior expertise [80,81,82] in analyzing cognitive processing EEG signals, we anticipated detecting variations specifically ion the designated cognitive electrodes. These electrodes are designated as optimal for observing cognitive activity according to the EGI 256-channel cap specifications. They were strategically positioned across the scalp, and they are sequentially numbered as follows: E98, E99, E100, E101, E108, E109, E110, E116, E117, E118, E119, E124, E125, E126, E127, E128, E129, E137, E138, E139, E140, E141, E149, E150, E151, and E152. The topographical map of these electrodes as places on the scalp can be found in Figure 1 in the EGI documentation [83,84].

The research protocol for both types of sessions is presented in Figure 1.

After the signal was recorded, we exposed it to low and high pass filtering, removed artefacts, and continued with the so-called interpolation of electrodes. Next, the signal was divided into 1 min long segments and Fourier transforms were applied to the calculation of the power spectrum densities (PSDs) to be averaged over this 1 min long time interval. Next, the data were divided into training and testing sets (80%/20%) and the classifier worked on the signals that it has never seen before.

During the mental task experiment, participants were asked to recall as many European country capitals, zodiac signs, and United States states from memory as possible. They were told that they would be asked to write down their responses after the experiment and that their reward depended on the results. It was assumed that this task would require mental effort, leading to a high level of mental workload and a stressful situation.

Initially, 60 participants were recruited from the students of Computer Science at Maria Curie-Sklodowska University in Lublin. These were all right-handed males aged 17 to 24, with an average age of 20.38 and a standard deviation of 1.52. Only men were chosen for the experiment because mainly male students of Computer Science attend the University where the research was conducted, and differences in electroencephalograms between men and women have been reported [85,86]. This was done to achieve a relatively equal cohort response.

It was ensured that the participants did not suffer from chronic diseases. They were asked to declare any serious diseases such as chronic fatigue syndrome, cancer, and other chronic diseases, including mental disorders, and if they did, they were automatically excluded from the cohort. The experimental cohort was divided into two sub-cohorts: A consisted of 30 subjects exposed to relaxation, and B consisted of 30 subjects asked to perform the mental task.

### 3.4. Inclusion and Exclusion Criteria

The inclusion criteria for the cohort in this experiment include being a short-haired, right-handed, healthy, Polish-speaking male between the ages of 17 and 24, with no history of chronic diseases, no current use of prescribed medication, soft drugs, or hard drugs, and the ability to attend study appointments with no technological requirements. Participants were also asked not to consume alcohol or any medication at least 72 h before participation in the experiment.

Exclusion criteria included being younger than 17 or older than 24 years, being left-handed, having long hair, not fluently speaking the Polish language, being seriously or chronically ill, currently taking prescribed medication, soft drugs, or hard drugs, having a medical treatment history in one year following the study, or being unable to attend study appointments. Participants who did not meet the inclusion criteria or declared any serious diseases, including mental disorders, were automatically excluded from the cohort. Prior to participating in the experiment, participants received information about EEG research and technology and signed an agreement for participation.

The proportion of women pursuing a computer science education remains low, making it challenging to create a well-balanced group for the experiment that included an equal number of left-handed and right-handed men and women. Additionally, it was observed that a significant majority of women studying computer science had long hair. It is worth mentioning that studies have documented variances in electroencephalogram readings between men and women [85,86], and we aimed to ensure a relatively equal response from the cohort.

### 3.5. The 14th min Choice Justification

In summary, the choice of the 14th min for analysis was based on a previous postulation that it is the most likely time for the participants to be experiencing a deep state of relaxation. To confirm this, the generalized linear model classifier (GLM) was used to distinguish between relaxation and mental state with an approximately 80% accuracy.

The generalized linear model enhances the general linear model by introducing a specified link function to establish a linear association between the dependent variable and the factors and covariates. The advantage over the general linear model is that there is no need for the data distribution to be normal. In the case of the presented research, the link function was logit. The dependent variable was the Mental Workload or Guided Imagery group. The factors were band (alpha, beta, and theta) powers from every minute of the recordings.

Generalized linear models (GLMs) are often used for time series analyses [87] and it is not aim of this paper to explain in detail all its cases and formulas. However, the idea of GLM consists of three components:

1. An exponential family of probability distribution (this means it is not necessary for a normal distribution);
2. A linear predictor η=Xβ;
3. A link function g such that $E\left(Y\right)=\mu =g^{-1}\left(\eta \right)$

where Y is the dependent variables vector (in our case GI/Mental task workload), E(Y) is the expected value of Y (it is either GI or MT), g is the so-called linking function (in our case *logit*), X is a matrix of the independent variables (in our case values collected from the EEG bands), and β represents model factors and is set by the model while training. In our case, the model is expressed by:

(1) g(E(Y))=Xβ

and g is a function expressed by:

(2) $$logit\left(p\right)=\sigma^{-1}\left(p\right)=ln\frac{p}{1-p}$$

for p∈(0,1).

**Figure 2:**
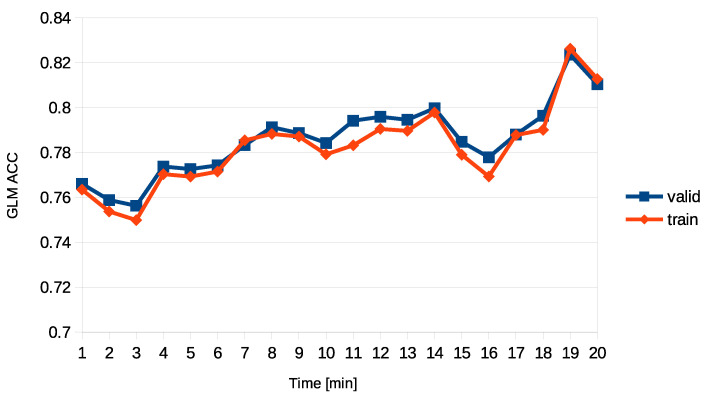
The 14th min choice justification.

To further validate the choice of the 14th min, the GLM accuracy was tested on each one-minute-long interval of time, from the beginning to the end of the recordings. The results showed a local maximum in the 14th min for both GI and Mental task sessions, followed by a falling slope until the 16th min. After the 17th min, the waking up process started, and the classifier’s accuracy increased, indicating a different and distinguishable state of brain activity. Therefore, the 14th min was chosen as the appropriate time for further analysis (Figure 2).

The intention behind using machine learning classifiers was to help in the classification of biomedical signals for therapy support [88,89]. These tools and algorithms have been used for a long time for the diagnosis of various disorders, such as alcoholism or depression [90,91]. Additionally, they have been used to measure various biological system behaviors and for diagnostic purposes [92]. Advanced modeling techniques have also been employed to better understand these systems [93,94]. The use of new measures, such as those defined in recent research [95,96], has further advanced the accuracy of these models.

### 3.6. The Final Cohort

Finally, after pre-processing the signal and eliminating the poor quality, as well as leaving only the participants who provided as with a full set of data and good EEG recordings and taking into account all the exclusion criteria, we had 20 subjects left in the GI sub-cohort and 28 subjects in the mental task engaged sub-cohort.

## 4. Statistical Analysis of the Data

The current study aimed to compare the effects of GI and a mental task intervention on cognitive and emotional measures, as well as to explore potential correlations between these measures. A group of participants were randomly assigned to either the GI or mental task group and completed a series of tests, including brain wave measures, attentional control tasks, and anxiety and affective measures.

Table 1 shows the participants’ characteristics for subjective measures in a study with two groups: the GI group (N = 20) and the mental task group (N = 28). The measures include anxiety, helplessness, stress reduction, and relaxation increase. A one-way analysis of variance (ANOVA) was conducted to test for significant differences between the groups.

In neuroscience research, longitudinal data are often analyzed using an analysis of variance (ANOVA) and a multivariate analysis of variance (MANOVA) for repeated measures (rmANOVA/rmMANOVA) [97]. MANOVA is an extension of ANOVA, which measures the impact of independent categorical variables upon numerous dependent continuous variables. It is a process used for comparing the sample means, which are multivariate in statistics. MANOVA is mostly used in a population with more than two variables. It is a non-parametric test. However, these analyses have special requirements: The variances of the differences between all possible pairs of within-subject conditions (i.e., levels of the independent variable) must be equal. They are also limited to fixed repeated time intervals and are sensitive to missing data [97]. In contrast, other models such as the generalized estimating equations (GEE) suggest another way to consider the data and the studied phenomenon. Instead of forcing the data into the ANOVAs assumptions, it is possible to design a flexible/personalized model according to the nature of the dependent variable.

We decided to use an ANOVA for our data analysis due to its balance and neuroscientific character.

For the anxiety measures (STAI Trait and STAI State) at pretest, there were no significant differences between the groups. For helplessness, there was also no significant difference between the groups. However, there was a significant difference in the stress reduction. An ANOVA showed a significant difference between the two groups (*p* < 0.05, $\eta^{2}$ = 0.102), indicating that the GI group had a greater reduction in stress levels compared to the mental task group. Finally, there was a marginally significant difference in the relaxation increase between the two groups, with the GI group showing a greater increase in relaxation.

The 14th min of the GI session was chosen for analysis using the general linear model (GLM) classifier because it was found to be the time when participants were in the deepest state of relaxation. The GLM was able to distinguish between relaxation and mental states with 80% accuracy [98], and the results showed that the 14th min had a local maximum for both GI and mental task sessions, making it an appropriate time for further analysis to find if a higher alpha power was significantly correlated with a better performance in attentional tests such as the numerical Stroop, anti-saccade, and Go/No-go tasks.

Table 2 presents the results of a study that compared two different interventions, GI and mental tasks, on brain wave patterns and attentional control measures.

The participants were 48 individuals, with 20 randomly assigned to the GI group and 28 to the mental task group. The following measures were collected for both groups: alpha power and Beta power brain wave activity at the 14th min of the intervention, attention control, numerical Stroop task (% errors), anti-saccade task (% errors), and Go/No-go task (% errors).

Table 2 presents the results of comparing the GI group and the mental task group in terms of brain waves and attentional control measures. The table includes the means and standard deviations of the alpha and Beta power in the 14th min of the GI and mental task groups, as well as the scores in the attention control measures. The ANOVA results include F-values, *p*-values, and effect sizes ($\eta^{2}$) for each measure. The results indicate a significant difference in the GI group, where we can observe a higher alpha power compared to the mental task group, which was statistically significant (F = 5.23, *p* = 0.023). However, there was no significant difference in beta power between the two groups. Referring to attentional control measures, the GI group had lower errors on the numerical Stroop task compared to the mental task group, and this difference was statistically significant (F = 8.06, *p* = 0.007, $\eta^{2}$ = 0.146). Similarly, the GI group had lower errors in the anti-saccade task compared to the mental task group, and this difference was also statistically significant (F = 7.31, *p* = 0.010, $\eta^{2}$ = 0.135). Although the GI group did not show significant improvements in the Go/No-go task, it is possible that this discrepancy can be explained by differences in the cognitive demands of the tasks. The Go/No-go task requires both response inhibition and working memory, whereas GI may not enhance the working memory to a sufficient degree.

The results suggest that GI may be more effective for enhancing attentional control in specific contexts, as it increases the alpha power and reduces stress levels through mental rehearsal and visualization, rather than through sustained focus practice like meditation.

The final analysis that was conducted in the described study is the Pearson’s R correlations, verifying the strength and direction of the relationships between different variables.

Table 3 presents the correlations between seven variables measured in the study. Variable 1 represents alpha power at the 14th min, while variables 2 and 3 represent errors in the numerical Stroop and anti-saccade tasks, respectively. Variable 4 represents stress reduction, variable 5 represents helplessness, and variables 6 and 7 represent the STAI Trait and STAI State anxiety measures, respectively. The correlation coefficients range from −1 to 1, with −1 indicating a perfect negative correlation, 0 indicating no correlation, and 1 indicating a perfect positive correlation. For example, the correlation between the alpha power and numerical Stroop error is −0.35, which indicates a negative correlation. As the alpha power increases, the numerical Stroop error tends to decrease.

The results indicate that there was a significant negative correlation between alpha power at the 14th min and errors on the numerical Stroop task and anti-saccade task, suggesting that a higher alpha power was associated with better performance in these tasks.

Additionally, there was a significant positive correlation between stress reductions and helplessness, indicating that higher levels of stress reduction were associated with lower levels of helplessness. Furthermore, the anxiety measures (STAI Trait and STAI State) were positively correlated with each other and with the anti-saccade task and the numerical Stroop task. This suggests that higher levels of anxiety were associated with poorer performances in these attentional control tasks. Notably, the correlation between the STAI State anxiety measure and the alpha power at the 14th min was also significant, indicating that a higher anxiety was associated with a lower alpha power. Overall, these findings highlight the complex relationships between brain wave activity, attentional control measures, stress reduction, helplessness, and anxiety. Further research is needed to better understand these relationships and their potential implications in cognitive functioning and mental health.

Two mediation models were employed to investigate how GI affects erroneous responses in the Stroop and anti-saccade tasks via alpha power at the 14th min. The findings indicate that alpha power at the 14th min acts as a dependable mediator between GI and the number of errors made in both attentional tasks, namely Stroop and anti-saccade tasks.

**Figure 3:**
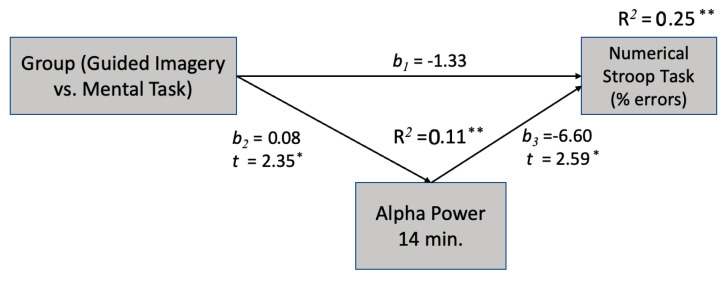
The effect of GI on reducing erroneous reactions in the Stroop test is mediated by the alpha power at 14 min. * *p* < 0.05, ** *p*< 0.01.

The mediation model (Figure 3) suggests that the relationship between GI and the Stroop test is mediated by the alpha power at the 14th min. Specifically, the significant negative coefficient between GI and the Stroop test suggests that GI leads to a better performance in the Stroop test and GI is a reliable mediator of the relationship.

**Figure 4:**
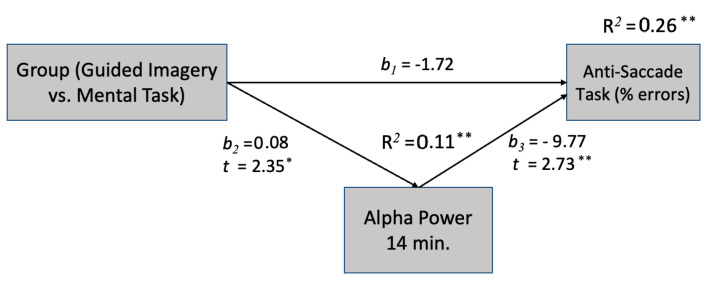
The effect of GI on reducing erroneous reactions in the anti-saccade test is mediated by the alpha power at 14 min. * *p* < 0.05, ** *p*< 0.01.

Based on a mediation analysis (Figure 4), the model suggests that the relationship between GI and errors in the anti-saccade test is partially explained by changes in the alpha power. A mediation analysis suggests that an increase in the alpha power is associated with a reduction in errors in the anti-saccade test.

The significance of the t-values indicates that the coefficients are unlikely to have occurred by chance, supporting the relationships between the variables in the mediation model. These results suggest that the use of GI may improve cognitive performance, particularly in tasks requiring inhibitory control, by increasing the alpha power. However, further research is needed to confirm these findings and explore the underlying mechanisms of this relationship.

## 5. Limitations of the Study

The study is subject to several limitations that should be considered in the interpretation of the findings. Firstly, the relatively small sample size employed in this study may constrain the generalizability of the results to larger populations or different demographic groups. Consequently, caution should be exercised when extrapolating the findings to broader contexts. Furthermore, the study primarily focused on healthy male participants with no prior experience with guided imagery sessions and no chronic medical conditions. Consequently, the extent to which the results can be applied to other populations or individuals with specific health conditions may be limited. Additionally, the study primarily examined the short-term effects of the guided imagery session, with limited investigations into the long-term or sustained benefits. Future research should address this limitation by investigating the durability of the observed effects over an extended period. It is worth considering for future studies the inclusion of an additional control group that receives either no intervention or an alternative intervention. The absence of such a control group in this study poses challenges in isolating the specific effects of guided imagery from other potential factors.

Taken together, these limitations underscore the need for future research with larger and more diverse samples, longer follow-up periods, and additional control groups. By addressing these methodological considerations, a more comprehensive understanding of the effectiveness and potential limitations of guided imagery can be achieved, not only in the context of stress management but also in terms of enhancing attentional control test results. Such investigations will provide valuable insights into the broader cognitive benefits of guided imagery and further enhance its potential as a therapeutic intervention.

## 6. Conclusions

This study investigated the effects of the GI relaxation technique on cognitive and emotional measures and explored potential correlations between these measures. Guided imagery offers a distinct experiential approach to mindfulness and mental well-being. While meditation primarily focuses on cultivating present-moment awareness and detachment from thoughts, guided imagery involves actively engaging the imagination to create vivid sensory experiences [99]. This approach can be particularly helpful for individuals who find it challenging to quieten the mind or those who benefit from more structured practices. A further exploration of guided imagery is worthwhile as it broadens our understanding of mindfulness, offers customization, and provides a complementary practice to enhance overall mental health [100]. The robust findings from this research provide compelling evidence supporting the efficacy of guided imagery (GI) as an intervention for stress reduction and relaxation, surpassing the effects observed in the mental task group. Notably, the GI group exhibited significantly higher levels of alpha power, a key indicator of brain wave activity associated with improved attentional control. The strong correlation between alpha power and enhanced performances in attentional tasks further reinforces the potential benefits of GI in optimizing cognitive functioning. These findings underscore the significance of incorporating the GI technique in stress management protocols and highlight its promising role in enhancing attentional control abilities. The findings obtained in this study align with the existing literature, providing consistent evidence that an increase in alpha power is associated with an improved performance in attentional tests. Moreover, the observed reduction in stress levels resulting from the guided imagery (GI) intervention contributes to enhanced attentional processes by mitigating the distraction caused by anxiety-related thoughts or worries. These results highlight the beneficial impact of GI on attentional functioning and support its potential as an effective strategy for optimizing cognitive performance in stress-inducing contexts.

Based on the findings of this study, the formulated hypotheses put forth by the researchers were supported. The guided imagery (GI) intervention resulted in an increase in alpha power and improved performances in attentional tests, specifically the Stroop and anti-saccade tasks. It is worth noting that the lack of significant improvements in the Go/No-go task can be attributed to the varying attentional demands across different tests. As previously described, these attentional tests assess distinct types of attentional control. For instance, the numerical Stroop task measures attentional inhibition, which involves suppressing irrelevant information and focusing on relevant stimuli. The anti-saccade task assesses attentional shifting, which pertains to the ability to shift attention from one target to another. On the other hand, the Go/No-go task evaluates attentional vigilance, which involves sustaining attention over time and responding selectively to relevant stimuli while ignoring irrelevant ones.

In contrast to mindfulness practices, GI does not enhance focused attention but rather involves the visualization of pleasant images which elicit stress- and anxiety-reducing responses, potentially influencing the alpha power. It is noteworthy that the alpha power has been found to be positively correlated with information processing speeds [101]. The results suggest that the GI intervention may have had a more pronounced effect on cognitive flexibility, which could have contributed to the improved performances in the Stroop and anti-saccade tasks. These findings highlight the unique cognitive mechanisms engaged during GI intervention and its potential to enhance cognitive flexibility in a manner distinct from traditional mindfulness practices. The mediation model examining the relationship between GI, alpha power at the 14th min, and performance on the Stroop and anti-saccade tests provides a comprehensive understanding of the interplay between these variables. It sheds light on the potential mechanisms through which GI can affect cognitive performance, particularly in the context of attentional control tasks. In summary, the mediation model presented here offers a valuable structure for comprehending the intricate associations between GI, alpha power, and cognitive performance. It underscores the necessity for additional investigations to gain a deeper understanding of this domain. In particular, pairwise comparisons methods (analyzed for accuracy by Koczkodaj [102]) can be considered.

In conclusion, this study offers valuable insights into the potential advantages of guided imagery (GI) as an intervention for enhancing cognitive performance and emotional well-being. The findings contribute to the expanding body of research on cognitive and emotional interventions, providing valuable knowledge that can inform the development of effective interventions targeting cognitive and emotional functioning. Further investigations are warranted to examine the long-term effects of GI interventions and delve deeper into the potential associations between these cognitive and emotional measures. Such research endeavors would help advance our understanding of the sustained effects and the intricate interplay between cognitive and emotional domains, ultimately contributing to the refinement of interventions aimed at promoting overall cognitive and emotional well-being.

Moreover, a notable feature of this research involved the application of multi-sensor EEG signal classification and a GLM for the categorization of two mental states. These findings offer compelling evidence regarding the potential for developing innovative therapies in the domain of human–machine interactions like in [103] and that EEG is not the only medium that can be used to support human–machine interaction control [104,105]. For instance, the study titled “Golden Subject Is Everyone: A Subject Transfer Neural Network for Motor Imagery-based Brain Computer Interfaces” [106] explores the use of neural networks to transfer knowledge between individuals in the context of motor-imagery-based brain–computer interfaces. The researchers propose a new approach that allows data from one participant to be used to train a neural network, which can then be applied to predict and interpret brain signals from a different participant. The findings indicate that this method has potential and could lead to the development of more inclusive and widely applicable brain–computer interfaces.

## Figures and Tables

**Table 1:** Participants’ characteristics for subjective measures. Bold means statistical significance.

	Guided Imagery Group (N = 20)		Mental Task Group (N = 28)		Statistical Test		
Measures	M	SD	M	SD	F	*p*	$\eta^{2}$
Anxiety measures (pre-test)							
STAI Trait	45.00	7.91	45.93	33,117	0.12	n.s.	n.s.
STAI State	39.85	9.98	40.29	31,959	0.15	n.s.	n.s.
Motivational and affective measures							
Helplessness (pre-test)	18.00	5.48	17.3	4.94	0.41	n.s.	n.s.
Stress reduction (before–after)	2.25	5.27	1.00	1.52	**5.12**	**0.03**	0.102
Relaxation increase (after–before)	2.25	5.17	1.15	2.67	2.28	0.14	0.048

**Table 2:** Participants’ characteristics for brain waves and attentional control measures. Bold means statistical significance.

	Guided Imagery Group (N = 20)		Mental Task Group (N = 28)		Statistical Test		
Measures	M	SD	M	SD	F	*p*	$\eta^{2}$
Brain waves							
Alpha power (14th min)	0.25	0.13	0.17	0.12	**5.23**	**0.023**	0.105
Beta power (14th min)	0.08	0.03	0.07	0.03	1.23	n.s.	n.s.
Attention control							
Numerical Stroop task (% errors)	1.35	1.92	3.24	2.51	**8.06**	**0.007**	0.146
Anti-saccade task (% errors)	1.87	3.16	4.42	3.16	**7.31**	**0.010**	0.135
Go/No-go task (% errors)	7.33	6.72	8.85	5.93	0.70	n.s.	n.s.

**Table 3:** Correlations between measures. Note: * *p* < 0.05, ** *p* < 0.01. Bold means statistical significance.

Variable	1	2	3	4	5	6	7
1. Alpha power 14 min	-						
2. Num. Stroop (% errors)	−0.35 **	-					
3. Anti-Saccade (% errors)	−0.45 **	−0.38 **	-				
4. Stress Reduction	**0.29** *	−0.03	−0.22	-			
5. Helplessness	0.24	−0.12	−0.04	**0.29** *	-		
6. STAI Trait	−0.12	0.10	0.27	0.10	**0.48** **	-	
7. STAI State	0.14	0.01	0.12	0.21	**0.37** **	**0.74** **	-

## Data Availability

The raw data supporting the conclusions of this manuscript will be made available by the authors without undue reservation to any qualified researcher.
